# Genomic Repeat Abundances Contain Phylogenetic Signal

**DOI:** 10.1093/sysbio/syu080

**Published:** 2014-09-25

**Authors:** Steven Dodsworth, Mark W. Chase, Laura J. Kelly, Ilia J. Leitch, Jiří Macas, Petr Novák, Mathieu Piednoël, Hanna Weiss-Schneeweiss, Andrew R. Leitch

**Affiliations:** ^1^School of Biological and Chemical Sciences, Queen Mary University of London, Mile End Road, London E1 4NS, UK; ^2^Jodrell Laboratory, Royal Botanic Gardens, Kew, Richmond, Surrey TW9 3DS, UK; ^3^School of Plant Biology, The University of Western Australia, Crawley WA 6009, Australia; ^4^Institute of Plant Molecular Biology, Biology Centre ASCR, Branišovská 31, České Budějovice, CZ-37005, Czech Republic; ^5^Systematic Botany and Mycology, University of Munich (LMU), Menzinger Straße 67, 80638 München, Germany; and ^6^Department of Systematic and Evolutionary Botany, University of Vienna, Rennweg 14, A-1030 Vienna, Austria

**Keywords:** Repetitive DNA, continuous characters, genomics, next-generation sequencing, phylogenetics, molecular systematics

## Abstract

A large proportion of genomic information, particularly repetitive elements, is usually ignored when researchers are using next-generation sequencing. Here we demonstrate the usefulness of this repetitive fraction in phylogenetic analyses, utilizing comparative graph-based clustering of next-generation sequence reads, which results in abundance estimates of different classes of genomic repeats. Phylogenetic trees are then inferred based on the genome-wide abundance of different repeat types treated as continuously varying characters; such repeats are scattered across chromosomes and in angiosperms can constitute a majority of nuclear genomic DNA. In six diverse examples, five angiosperms and one insect, this method provides generally well-supported relationships at interspecific and intergeneric levels that agree with results from more standard phylogenetic analyses of commonly used markers. We propose that this methodology may prove especially useful in groups where there is little genetic differentiation in standard phylogenetic markers. At the same time as providing data for phylogenetic inference, this method additionally yields a wealth of data for comparative studies of genome evolution.

Understanding aspects of comparative evolution, including at its simplest relationships between taxa at varying levels of classification, is being revolutionized by the advent of next-generation sequencing (NGS) technologies. All recent methods in this area are based on multiplexing samples from diverse taxa, thereby maximizing the number of taxa that can be sequenced in one lane or one plate of an NGS run (e.g., Illumina). NGS approaches have enabled a quantum leap in the amount of data available while becoming increasingly cost-effective (Glenn 2011). Approaches include amplicon sequencing (sequencing of specific genes or regions of interest) using barcoded primers (Meyer et al. 2007; Bybee et al. 2011), full mitochondrial and plastid genome sequencing (Timmermans et al. 2010; Barrett et al. 2013; Straub et al. 2012; Kayal et al. 2013), and phylogenomics based on the full complement of protein-coding genes (Zhou et al. 2012; Yoder et al. 2013). Many recent approaches are based on reduced-representation libraries (i.e., reducing genomic complexity/increasing recovery of homologous regions across taxa); in this arena RAD-sequencing based on restriction-site associated DNA fragments scattered across the genome (Rubin et al. 2012; Wagner et al. 2012) and hybridization methods of targeted capture, so-called “pull-down” approaches (Cronn et al. 2012; Carpenter et al. 2013; Guschanski et al. 2013), are two of the most common methodologies.

However, in such phylogenetic/phylogenomics studies, and indeed in broader studies of comparative evolution, the repetitive portion of the genome is often discarded without consideration of any potential use. Repetitive elements in genomes consist of both tandem repeats and interspersed mobile elements (e.g., DNA transposons and retrotransposons). In angiosperms (flowering plants), such repeats are diverse and numerous, contributing up to 70%–80% of nuclear genomic DNA (gDNA), thus making flowering plants an excellent group in which to study the dynamics of repetitive element evolution (Hansen and Heslop-Harrison 2004; Wicker et al. 2007; Leitch and Leitch 2008; Kelly et al. 2012). Genome sizes vary 2400-fold in angiosperms alone (Pellicer et al. 2010; Kelly and Leitch 2011); aside from cases involving wholegenome duplication, much of this variability can be explained by differing amounts of repetitive DNA.

NGS of a small, random sample of the genome (0.5–5% genome proportion [GP], i.e., genome coverage as a percentage) results in data consisting mainly of repetitive sequences; genic regions will not be adequately covered in such a dataset, but repeats present in thousands of copies will be well represented. Previous analyses have shown that low-coverage sequencing of gDNA, followed by graph-based clustering of sequence reads, is sufficient to provide characterization of many hundreds or thousands of well-represented repeats (Macas et al. 2007; Novak et al. 2010; Renny-Byfield et al. 2011); these studies also provide detailed insights into patterns of genome evolution (Renny-Byfield et al. 2011; Piednoël et al. 2012; Leitch and Leitch 2012; Renny-Byfield et al. 2013). Low-coverage gDNA sequencing (i.e., “genome skimming”; Straub et al. 2012) and repeat clustering are now both cost-effective and easy to implement (Novak et al. 2013). The proportion of sequence reads representing a particular repetitive element cluster has also been shown to accurately reflect genomic abundance (Macas et al. 2007; Novak et al. 2010; Renny-Byfield et al. 2011; 2012). Repetitive elements are scattered across the genome and provide much of the characteristic differences between chromosomes and chromosomal subregions, including those in which the majority of genes are embedded (Brookfield 2005). Thus, relative abundance of well-represented repeats is reflective of broad-scale genome composition. Localization of repeats on chromosomes and use of repeats as markers for fluorescence *in-situ* hybridization (FISH) in some groups has shown that often the most-parsimonious explanation for these localizations and rearrangements reflect hypotheses of the species tree derived from other data types, usually DNA sequence data (e.g., Lim et al. 2000; 2006). In structure and chromosomal position, repeats in closely related species are nearly identical, whereas more distantly related species diverge in repeat structure and location as genetic similarity decreases.

Here we test the usefulness of a novel phylogenetic methodology based on the abundance of different repetitive elements, estimated through bioinformatic analysis of NGS reads from a small proportion of the genome. Previously similar studies have found that genomic signatures present in the frequency of short sequence repeats can be used to reconstruct phylogenetic relationships, i.e., tetranucleotide frequencies in microbial genomes (Pride et al. 2003) and 2- to 5-nt repeats in birds (Edwards et al. 2002). Here we use different criteria and a clustering method in order to identify homologous repeat classes. This method can essentially be viewed as a hybrid between molecular systematics and morphometric cladistics, as abundances of repetitive DNAs are used as continuously varying characters for phylogenetic inference. We utilize in combination graph-based clustering estimation of repeats (Novak et al. 2010) and the computational methodology of Goloboff et al. (2006) in particular, which allows for analysis of continuous characters without assignment (coding) of arbitrarily circumscribed characters, implemented in the software “tree analysis using new technology,” TNT (Goloboff et al. 2003a, 2008). Such a combined approach has been utilized successfully with eigenshape-based geometric morphometrics and continuous character phylogenetics in TNT (Smith and Hendricks 2013). We investigate this method in six diverse groups – five orders of angiosperms and one insect group, with differing genome sizes and amounts of repetitive DNA, and show a high (but not always identical) level of congruence with previously hypothesized species trees (i.e., current knowledge from gene trees and morphological circumscriptions) at a variety of taxonomic levels.

## Materials and Methods

### Tissue Sources and High-throughput Sequencing of gDNA

#### Nicotiana

Plant materials (accession numbers), DNA extraction and Illumina sequencing details (including NCBI Short Read Archive [SRA] accession numbers) can be found in Renny-Byfield et al. (2012) and Renny-Byfield et al. (2013).

#### Orobanchaceae

Plant materials (including voucher specimen details), DNA extraction and 454 sequencing details (including SRA accession numbers) for this dataset can be found in Piednoël et al. (2012). Orobanchaceae is the largest family of parasitic flowering plants. Four genera were included in our dataset, representing a variety of life history strategies: *Lindenbergia*, autotrophic, nonparasitic; *Schwalbea*, parasitic but still photosynthetic; four species of *Orobanche*, nonphotosynthetic, parasitic, including one tetraploid species (*O. gracilis*); and three species of *Phelipanche*, nonphotosynthetic, parasitic.

#### Fabeae

Seeds of *Vicia tetrasperma* (VIC726), *V. hirsuta* (VIC728), *V. sylvatica* (VIC63), and *V. ervilia* (ERV52) were obtained from the seed bank of the Leibniz Institute of Plant Genetics and Crop Plant Research (IPK), Germany. Seeds of *Lathyrus sativus* and *L. latifolius* were purchased from Fratelli Ingegnoli S.p.A., Milano, Italy (cat.no. 455) and SEMO Smrzice, Czech Republic (acc.no. 1-0040-68867-01), respectively. *Lathyrus vernus* was collected from a wild population at Vidov, Czech Republic (GPS 48°55'17.401”N, 14°29'44.158″E). *Pisum fulvum* (accession ICARDA IG64207) was provided by Petr Smykal, Palacky University, Olomouc, Czech Republic. In all species, genomic DNA was extracted from isolated leaf nuclei (Macas et al. 2007) and sequenced on the Illumina platform (paired-end 100 nt reads) at Elim Biopharmaceuticals, Hayward, USA (*P. fulvum*) or GATC Biotech, Konstanz, Germany (all other species). Illumina sequencing of *P. sativum* was described in Neumann et al. (2012). Voucher specimens are available for all material sequenced at IPMB, CZ. All read data are available at the SRA with the following accession numbers: *V. hirsuta—*ERR413114; *V. ervilia—*ERR413112; *V. sylvatica—*ERR413113; *V. tetrasperma*—ERR413111; *Lathyrus sativus*—ERR413118 & ERR413119; *L. vernus—*ERR413116 & ERR413117; *L. latifolius—*ERR413120; *Pisum sativum*—ERR063464; *P. fulvum—*ERR413083. Tribe Fabeae Rchb. is a group of five genera and ∼380 species, containing several important crop species including pea (*Pisum sativum*). In our analysis we included species from three genera, although this includes species proposed to be members of a further two new genera (Schaefer et al. 2012).

#### Fritillaria

DNA extractions were sourced from the Royal Botanic Gardens, Kew DNA Bank (http://apps.kew.org/dnabank.homepage.html). At the University of Liverpool, 454 sequencing was performed by the Centre for Genomic Research; reads were trimmed to 100 bp prior to clustering, and any reads of < 100 bp were discarded. All read data are available at the SRA with the following accession numbers: *F. affinis—*ERR571997; *F. alfredae* subsp. *glaucoviridis—*ERR571998; *F. davidii*—ERR571999; *F. imperialis—*ERR572000; *F. koidzumiana—*ERR572001; *F. maximowiczii—*ERR572002; *F. pluriflora—*ERR572003; *F. sewerzowii—*ERR572004; *F. tubiformis—*ERR572005; *Lilium pyrenaicum—*ERR572006.

*Fritillaria* is a genus of bulb-bearing petaloid monocots with species possessing some of the largest recorded genome sizes (Ambrozova et al. 2011; Kelly and Leitch 2011). It comprises approximately 140 species (Rix 2001) and is closely related to the genus *Lilium*. In our analysis, we had nine representatives of the genus from each of the two main clades—the North American and the Eurasian clades (Rønsted et al. 2005; Kelly and Leitch 2011).

#### Drosophila

Illumina reads for the following species were downloaded from the SRA: *Drosophila bipectinata—*SRR345542; *Drosophila suzukii—*SRR1002946; *Drosophila biarmipes—*SRR345536; *Drosophila ananassae—*SRR491410; *Drosophila melanogaster—*SRR1005465; *Drosophila sechellia—*SRR869587; *Drosophila simulans—*SRR580369.

#### Asclepias

Illumina reads for species from the Sonoran Desert clade of *Asclepias* were downloaded from the SRA: *A. macrotis* 149—SRX384308; *A. albicans* × *subulata* 282—SRX384307; *A. cutleri* 382—SRX384306; *A. subulata* 423—SRX384305; *A. macrotis* 150—SRX384304; *A. albicans* 422—SRX384303; *A. subulata* 411—SRX384302; *A. masonii* 154—SRX384301; *A. leptopus* 137—SRX384300; *A. cutleri* 421—SRX384299; *A. coulteri* 45—SRX384298; *A. subaphylla* 272—SRX384297; *A. subaphylla* 271—SRX384296; *A. albicans* 003—SRX384295; *A. syriaca* 4885—SRX040889.

Further details of raw data, quality filtering and resultant read datasets for all examples are provided in Online Appendix 1 (http://dx.doi.org/10.5061/dryad.vn0gc).

### Genome Size Estimation

In order to calculate the number of reads for each comparative clustering an accurate genome size should ideally be available for each species. For most datasets genome sizes were available from the Plant DNA C-Values database (http://data.kew.org/cvalues/) or were estimated using flow cytometry. For *Drosophila*, genome sizes were taken from the Animal Genome Size database (http://www.genomesize.com). For *Asclepias* genome sizes were assumed to be equal (423 Mb, as for *A. syriaca*; Bai et al. 2012), as data were unavailable for each species. Without accounting for genome size (e.g., taking the same number of reads), the abundance of each cluster is more likely to reflect genome size rather than the proportion of that repeat in the genome. Genome size ranges (1C) in each dataset are as follows: *Nicotiana* (1.51–5.32 Gb); Orobanchaceae (0.454.32 Gb); Fabeae (3.05–9.98 Gb); *Fritillaria* (30.1–75.7 Gb); *Asclepias* (n/a); *Drosophila* (0.16–0.20 Gb).

### Clustering of Repetitive DNA

Graph-based clustering of NGS reads was performed as described in Novak et al. (2010) using the latest Galaxy-based web server implementation of the pipeline, RepeatExplorer (Novak et al. 2013). In brief, all sequence reads (sequence data) are subjected to pair-wise (BLAST) comparison, and similarities are represented by a graph structure in which nodes represent sequence reads and overlapping reads are connected by edges. Edge weights represent the amount of sequence similarity (similarity scores). Clusters of nodes more frequently connected to one another than to outside nodes in the graph represent families of genomic repeats or their parts. Families would be likely to include sequences of the same length (or portions thereof) in which sequence variation is low, 90% similarity over at least 55% of their length.

Combined datasets of reads (sequence data) were compiled as follows: (1) 5% genome proportion (GP) each of four diploid species of *Nicotiana* L. (*N. sylvestris* Spreng., *N. tomentosiformis* Goodsp., *N. attenuata* Torr., *N. obtusifolia* M.Martens & Galeotti); (2) 5% GP each of the four diploid species of *Nicotiana* in (1) and two species of allopolyploid section *Repandae* (*N. repanda* Sims and *N. nudicaulis* S.Watson); (3) 5% GP each of the four diploid species of *Nicotiana* in (1) and two types of *N. tabacum* L. (*N. tabacum* SR1A and *N. tabacum* TR1A synthetic); (4) 2.08% GP each of nine species of Orobanchaceae (*Lindenbergia philippensis* (Cham. and Schltd.) Benth., *Schwalbea americana* L., *Phelipanche ramosa* (L.) Pomel, *Phelipanche purpurea* (Jacq.) Soják, *Phelipanche lavandulacea* Pomel, *Orobanche pancicii* Beck, *Orobanche gracilis* Beck, *Orobanche cumana* Wallr., *Orobanche crenata* Forssk.); (5) 1% GP each of nine species of tribe Fabeae (*Vicia sylvatica* L., *Vicia ervilia* Willd., *Vicia hirsuta* (L.) Gray, *Vicia tetrasperma* (L.) Schreb., *Pisum sativum* L., *Pisum fulvum* Sibth. & Sm., *Lathyrus sativus* L., *Lathyrus vernus* (L.) Bernh., *Lathyrus latifolius* L.); (6) 0.01% GP each of nine species of *Fritillaria* L. and one of *Lilium* L. (*Fritillaria affinis* (Schult. & Schult.f.) Sealy, *F. alfredae* subsp. *glaucoviridis* (Turrill) Rix, *F. davidii* Franch., *F. imperialis* L., *F. koidzumiana* Ohwi, *F. maximowiczii* Freyn, *F. pluriflora* Torr. ex Benth., *F. sewerzowii* Regel, *F. tubiformis* Gren. & Godr., *Lilium pyrenaicum* Gouan); (7) 2% GP each of 15 *Asclepias* L. (*Asclepias syriaca* L. 4885, *A. albicans* S. Watson 003, *A. albicans* 422, *A. coulteri* A. Gray 45, *A. cutleri* Woodson 382, *A. cutleri* 421, *A. leptopus* I. M. Johnst. 137, *A. macrotis* Torr. 149, *A. macrotis* 150, *A. masonii* Woodson 154, *A. subaphylla* Woodson 271, *A. subaphylla* 272, *A. subulata* Decne. 411, *A. subulata* 423, *A. albicans* × *subulata* 282; (8) 5% GP each of 7 *Drosophila* species (*D. ananassae*, *D. bipectinata*, *D. suzukii*, *D. biarmipes*, *D. melanogaster*, *D. sechellia*, *D. simulans*). Different GP values were used across datasets due to genome size differences and the amount of sequencing data available or that could be clustered with the available computing power.

Separate comparative analyses (i.e., simultaneous clustering of reads from all species in the dataset) were run for each dataset on RepeatExplorer (Novak et al. 2013), using default settings (i.e., similarity threshold of 90% over 55% of the read length). Reads were prefixed with codes specific to the taxon in question, enabling comparative analysis of repetitive element abundances in different taxa. Comparative counts of the number of reads in each cluster (which is proportional to their genomic abundance) were used for phylogenetic analyses. Plastid and mitochondrial reads were either filtered out prior to clustering (using BLAST and custom scripts) or were identified after clustering (BLAST to most closely related plastome currently available) and plastid clusters removed prior to phylogenetic inference.

### Homology of Repetitive DNA Clusters

The extent to which clusters represent homologous entities is dictated by the similarity parameters specified. We use the default settings of RepeatExplorer, with a threshold similarity of 90% over 55% of the read length to be exceeded in order for a hit to be recorded. Clusters are then produced using a graph-based algorithm and a principle of maximum modularity, which results in clusters where most reads have a high similarity to one another within clusters and a low similarity between clusters (see Novak et al. [2010] for further details on the clustering process). Different repeats will form different clusters in the output of RepeatExplorer and are treated here as separate evolutionary entities (characters). The abundance of a repeat in a species, its genome proportion, depends on repeat copy number and genome size. Tandem repeats have variable monomer sizes up to 180 bp; those with monomer sizes shorter than the read length (typically 100 bp) will form a spherical graph, and those with monomer sizes greater than the read length will form a ring graph structure.

Plant genomes in particular contain a large abundance of LTR retroelements (LTR-REs), which are typically several kb, up to 5kb. These repetitive elements are complex, often dispersed across the genome, and there may be a spectrum of related (or degraded products) of similar LTR retroelements. Based on the RepeatExplorer threshold and graphical algorithm, LTR-REs are often split into different clusters, as parts of these elements are less conserved (e.g., around the LTR) than others (e.g., the protein-coding domains). Sequence divergence within the protein-coding domains is insufficient for phylogenetic analysis, although the number of elements is variable and putatively indicative of evolutionary history. Although LTR-REs may be split over several clusters, they will be split in the same way for every species included in the same clustering run, thereby preserving phylogenetic signal, and each piece of LTR-RE would be expected to contain a uniform phylogenetic pattern.

### Assembly of High-copy DNA Sequences

High-copy DNA sequences were assembled directly from short read data using the program MIRA (http://www.chevreux.org/projects_mira.html). The general settings used in the manifest file are provided in Online Appendix 1. The following assemblies were performed: (2) *Nicotiana*—large subunit rDNA and whole plastomes were assembled by mapping to *Nicotiana tabacum* sequences as a reference, using raw Illumina reads; (2) *Fritillaria*—whole plastome sequences were assembled directly from plastid 454 reads only (filtered using a custom perl script and BLAST), using the *Lilium longiflorum* plastid genome as a reference; (3) Orobanchaceae—whole plastomes for *O. cumana*, *O. pancicii*, *O. crenata* were assembled from raw 454 reads using the *O. gracilis* plastome as a reference and *P. lavandulacea* was assembled using *P. ramosa* as a reference.

### Phylogenetic Analyses

#### Maximum parsimony analysis

Data matrices consisting of the 1000 most abundant clusters, each representing a repetitive element family, were converted to legal TNT format (modified Hennig86). All abundances were transformed by a constant factor dependent upon the largest cluster abundance in the matrix. Each abundance was divided by this factor (factor=largest abundance/65) in order to make all numbers in the matrices ≤ 65, the maximal value for continuous character implementation in TNT tree searches (Goloboff et al. 2003a; 2006; 2008). This factorial transformation does not affect the normal distribution of abundance for each cluster and is only necessary for efficient implementation in the TNT program, as described below.

Trees were inferred using maximum parsimony (MP), utilizing the implementation of Farris' algorithm for the down-pass and Goloboff's algorithm for the up-pass, as described in Goloboff et al. (2006). In such an approach, continuous characters are not arbitrarily recoded but are simply used as additive characters (i.e., count changes can be of noninteger differences). Implicit enumeration (branch and bound) tree searches were used for datasets in this study owing to the small number of taxa in each dataset. Resampling was performed using 100 000 replicates and symmetrical resampling, a modification of the standard bootstrap (Goloboff et al. 2003b). Sequence trees were inferred using the same method for comparison, with gaps coded as missing data. The same phylogenetic reconstruction methodology was employed to enable direct comparison to the repeat trees.

The following datasets were used: (1) full plastomes and 18S-5.8S-26S rDNA for *Nicotiana* diploids and section *Repandae* (assembled); (2) full plastomes for *Fritillaria* (assembled); (3) combined matrix of 17 mitochondrial and nuclear genes for *Drosophila* (28S, *adh*, *amy*, *amr*, *cdc6*, *COI*, *COII*, *ddc*, *esc*, *gpd*, *h2s*, *hb*, ITS, *ND1*, *ND4*, *nup* and *ptc* – see Yang et al. (2012) for GenBank accession numbers); (4) whole plastomes and complete 26S to 18S rDNA cistron for *Asclepias*—alignments taken from Straub et al. (2012); (5) whole plastomes for Orobanchaceae (assembled); and (6) nuclear ITS rDNA and plastid *trnL* from Schaefer et al. (2012) for tribe Fabeae.

#### Maximum likelihood analysis

Maximum likelihood (ML) trees were computed using gene frequency/continuous character implementation in Contml, part of the Phylip package (Felsenstein 1989; 2005). This method assumes that each character evolves independently and only in accordance with random genetic drift, using a Brownian motion model of likelihood. Matrices were transformed such that cluster abundances represented allele frequencies (0–1) by dividing all clusters by the largest cluster size; this is required for resampling prior to ML tree computation. Resampling from the matrix with replacement (bootstrapping) was first carried out using Seqboot for 1000 replicate datasets. ML analyses were then performed on all 1000 datasets using Contml, and bootstrap percentages mapped onto the strict consensus tree for each dataset computed using Consense.

All trees were viewed in FigTree (http://tree.bio.ed.ac.uk/software/figtree/) and further edited in iDraw (Indeeo, Inc.). All ML trees are shown in Online Appendix 2. Reticulation in the *Nicotiana tabacum* dataset was explored using SplitsTree4 (Huson and Bryant 2006), using 10 000 bootstrap trees from the MP analysis as input for filtered supernetwork analysis (filtering performed at 10% of all input trees). *Nicotiana tabacum* is a relatively recently formed allotetraploid; its two parents have been determined to be *N. tomentosiformis* and *N. sylvestris* (Chase et al. 2003).

### Testing Method Performance

**Figure 1.**
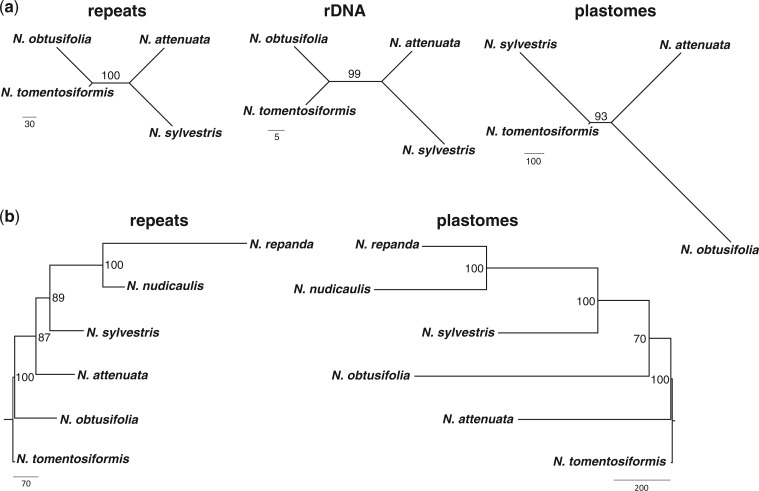
Phylogenetic relationships in *Nicotiana* (Solanaceae). a) Unrooted most parsimonious trees for repeats, large rDNA subunit sequences, and plastome sequences for four diploid *Nicotiana* taxa. b) Repeat and plastome trees including diploids from a) and *Nicotiana* section *Repandae* (*N. nudicaulis* and *N. repanda*). Repeat trees are based on 1000 cluster abundances from 5% genome proportion clustering. Maximum parsimony analysis with 10 000 symmetric bootstrap replications and bootstrap percentages plotted onto the single most parsimonious tree in each case. Numbers on nodes represent BPs≥50; branch lengths are shown from the single MPT and scale bars at the bottom left and right show relative numbers of step changes.

To test performance of the method several parameters were analyzed with the smallest clustering dataset (four diploid species of *Nicotiana*) and in TNT as above, but with modifications described below. In each case, the resultant tree was compared with the expected tree topology (Fig. 1a) and the symmetric bootstrap percentage recorded.

#### Reproducibility and relationship with genome proportion

To find the minimal GP necessary to resolve relationships, several sequence datasets were produced at 11 levels of GP from 0.005% to 5.120% (doubling of GP at each step). Three replicate clustering runs were computed for each GP. The mean support and standard error were calculated and used, in addition to tree support and topology, to observe how reproducibility varies with GP.

#### Relationship between phylogenetic signal and cluster number

To evaluate the number of characters (clusters) sufficient to resolve the tree, trees were built with different numbers of clusters, varying from 5 to 1000 for 25 datasets, and the tree inferred each time, comparing the topology and support percentages.

#### Variance in phylogenetic signal across the matrix

Variance in phylogenetic signal across the cluster abundance matrix was tested by partitioning it into sets of 150 cluster abundances (this number chosen from the cluster number analysis above). Trees were then inferred from each set, and the resulting resolution and symmetric bootstrap support of the unrooted tree were then estimated. Three GPs were tested (2.00%, 0.32%, and 0.07%) in order to show how different partitions respond at different GPs (chosen to represent difference of three orders of magnitude).

#### Effect of sampling – range analysis

To evaluate the effect of sampling sequence data on tree building, trees were inferred from clustering of three random samplings of read data. The mean and its standard error were calculated for abundance of each cluster. A phylogenetic analysis based on the range of the mean ±1 standard error of the mean was conducted, as ranges may more accurately reflect the phylogenetic signal in continuous characters (Goloboff et al. 2006), thereby reducing the artefact of two taxa appearing as distinct when they are not. Clusters (repetitive element abundances) that have overlapping normal distributions result in a step count of 0, i.e., no change. Range analysis was tested with a GP of 0.32%.

#### Phylogenetic informativeness of repeat types

The relative informativeness of different repeat types was analyzed by creating subsets of the original matrix based on different repeat annotations. Annotations were assigned to the following categories based on BLAST hits to repbase, custom-protein domain database in the RepeatExplorer pipeline and graph structure: DNA transposon, Ty1/Copia LTR retrotransposon, Ty3/Gypsy LTR retrotransposon, rDNA, satellite, and other (e.g., nonLTR retrotransposon) including unclassified repeats. Matrices were created based on each repeat type, for each example taxon dataset, and trees were inferred as above. The mean bootstrap (as a proxy for tree resolution) was computed for each analysis (Fig. 5).

## Results

### *Example 1*—Nicotiana (*Solanaceae; Solanales*)

The unrooted tree presented in Figure 1a contains four diploid species of *Nicotiana* and mirrors gene trees based on other nuclear DNA regions (Chase et al. 2003; Clarkson et al. 2004; 2010; Kelly et al. 2013), including rDNA sequences reconstructed from the NGS data (Fig. 1). There is a different relationship for these four using the plastome tree constructed from these NGS data (Fig. 1a), in line with plastid gene trees published previously (Clarkson et al. 2004).

In a further analysis of *Nicotiana*, tree resolution and topology were investigated using data from diploid species and allotetraploid species from two sections of *Nicotiana* (*Nicotiana* sections *Repandae* and *Nicotiana*). *Nicotiana* section *Repandae* is a group of four allopolyploids derived from a single allopolyploid formation event approximately 5 Ma (Clarkson et al. 2005; Parisod et al. 2012; Renny-Byfield et al. 2013). Genomes of sect. *Repandae* have experienced extensive genome turnover subsequent to their formation, and the genomes retain more similarity to the extant relative of their maternal progenitor, *N. sylvestris*, rather than the extant relative of their paternal progenitor, *N. obtusifolia* (Chase et al. 2003; Clarkson et al. 2004; 2005; 2010; Parisod et al. 2012; Kelly et al. 2013; Renny-Byfield et al. 2013). This striking bias is supported in our analysis here (Fig. 1b), in which *N. repanda* and *N. nudicaulis* together (100 bpP) are strongly supported as sister (89 bp) to *N. sylvestris*. Previous analyses of repetitive DNA and genomic *in situ* hybridization (GISH) have shown that the genomes of sect. *Repandae* have diverged extensively since their formation, despite being each other's closest relatives, through loss of middle and lower-abundance repetitive elements (Renny-Byfield et al. 2013). This is particularly evident in *N. repanda* (Fig. 1b).

**Figure 2.**
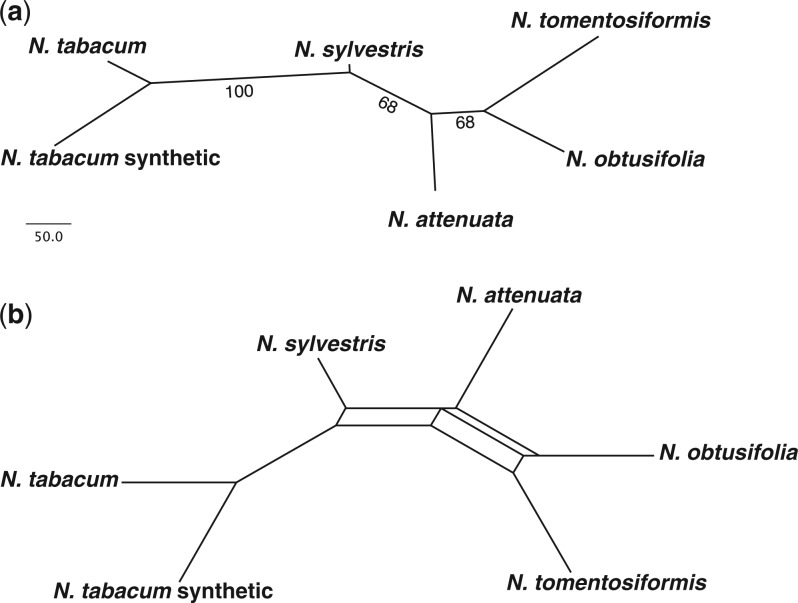
Phylogenetic relationships in a young allopolyploid, *Nicotiana* section *Nicotiana* (*N. tabacum*) and related diploid progenitor taxa (Solanaceae). a) Unrooted most parsimonious tree for repeats based on 1000 cluster abundances from 5% genome proportion clustering, maximum parsimony analysis with 10 000 symmetric bootstrap replications and bootstrap percentages plotted onto the single MPT. b) Filtered supernetwork showing relationships present in 10% of the bootstrap trees from a). Numbers on nodes represent BPs≥50; branch lengths are shown from the single MPT. The supernetwork is presented in order to present conflicting splits present due to recent reticulation.

To contrast this example, we examined how the method performs with a different allopolyploid section of much more recent origin—*Nicotiana* sect. *Nicotiana*, which contains the familiar allotetraploid *Nicotiana tabacum*, the most common tobacco species in commerce. *Nicotiana tabacum* is estimated to have originated 200 000 years ago or less, and its formation involved entities closely related to extant *N. tomentosiformis* and *N. sylvestris*, its paternal and maternal progenitors, respectively (Chase et al. 2003; Clarkson et al. 2004). GISH is able to distinguish the progenitor genomes (Chase et al. 2003), the T-genome from *N. tomentosiformis* and the S-genome from *N. sylvestris*. Analyses of repetitive DNA show the genome of *N. tabacum* has preferentially lost paternal repeats and is much more similar to *N. sylvestris* (Renny-Byfield et al. 2011; 2012), although its nrITS and IGS sequences of ribosomal DNA are identical to its paternal progenitor, *N. tomentosiformis* (Chase et al. 2003; Kovarik et al. 2012). In repeat phylogenetic analyses the tree with all four diploid species and *N. tabacum* shows that *N. tabacum* is more closely related to *N. sylvestris* than to *N. tomentosiformis* (Fig. 2a), reflecting that abundances of repetitive DNA in *N. tabacum* are in general more similar to those in the maternal parent, *N. sylvestris* (Fig. 2a). This result was previously found based on analyses of the GP in different clusters of repetitive DNA, which showed a preferential loss of paternal (i.e., *N. tomentosiformis*) repeats in *N. tabacum* (Renny-Byfield et al. 2011; 2012). Nevertheless, the supernetwork (Fig. 2b) illustrates the presence of splits that group *N. tabacum* with *N. tomentosiformis* in addition to placing it with *N. sylvestris*, indicating that some repeats inherited from the paternal progenitor are still present. Analysis of 10 000 bootstrap trees reveals that *N. tabacum* groups with *N. tomentosiformis* in 17% of the trees; in the remaining 83% of trees it is sister to *N. sylvestris*. Additionally, the relatively long branch length separating the *N. tabacum* samples from that of *N. sylvestris* highlights the retention of characters conflicting with its position as sister to *N. sylvestris* (i.e., presence of paternal-type repeats).

### *Example 2*—Fritillaria (*Liliaceae; Liliales*)

**Figure 3.**
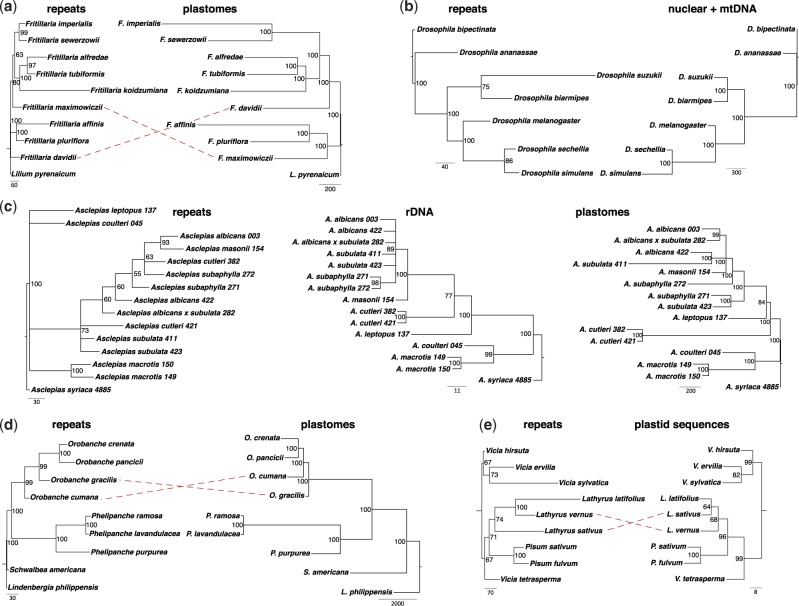
Phylogenetic relationships in: a) *Fritillaria* (Liliaceae). Trees for repeats and plastome sequences are shown; repeat tree based on 1000 cluster abundances from 0.01% genome proportion clustering. b) *Drosophila*, the *melanogaster* species group (Drosophilidae). Trees for repeats and combined matrix of 17 nuclear and mitochondrial genes (see methods for full details); repeat tree based on 1000 cluster abundances from 5% genome proportion clustering. c) The Sonoran Desert clade of *Asclepias* (Apocynaceae). Trees for repeats, 26S to 18S complete rDNA cistron sequences and plastome sequences are shown; repeat tree based on 1000 cluster abundances from 2% genome proportion clustering (assuming the same genome size of 420 MBp in each—see methods). d) Orobanchaceae. Repeat tree and plastome tree shown; repeat tree based on 290 cluster abundances from 2% genome proportion clustering. e) Fabeae (Fabaceae). Repeat tree and tree based on combined plastid *trnL*/nuclear ITS shown; repeat tree based on 1000 cluster abundances from 1% genome proportion clustering. Maximum parsimony analysis with 10 000 symmetric bootstrap replications and bootstrap percentages plotted onto the single most parsimonious tree in each case. Numbers on nodes represent BPs≥50; branch lengths are shown from the single MPT and scale bars at the bottom left and right show relative numbers of changes. Dashed lines show instances of incongruence between repeat trees and DNA sequence trees.

**Figure 4.**
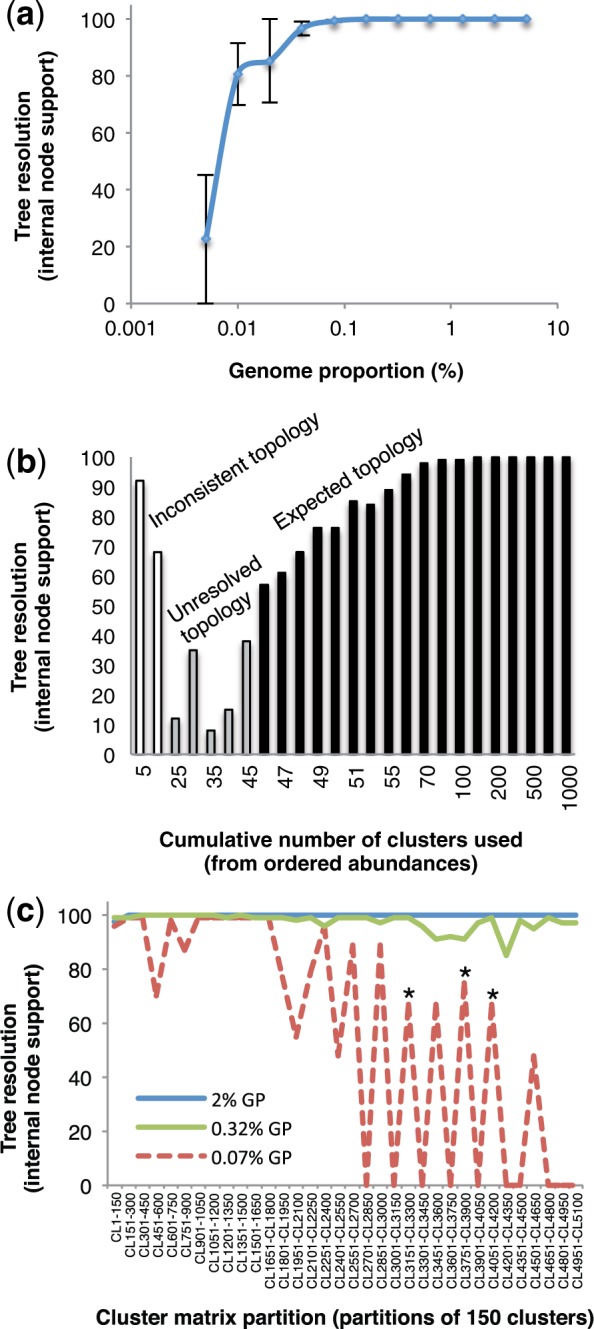
Performance measures using the four-taxon diploid *Nicotiana* dataset. a) Analysis of genome proportion (GP%) vs. tree support as the symmetric bootstrap of the unrooted tree. b) Analysis of total number of clusters used vs. tree support as the symmetric bootstrap. c) Partition analysis of 150-cluster segments of the dataset at three levels of GP: 2%, 0.32%, and 0.07%. Asterisks in c) represent trees that contain inconsistent species groupings.

*Lilium* is the designated outgroup, following Rønsted et al. (2005), and our analysis generally places species into their expected clades (Fig. 3a). Other than the placement of *F. maximowiczii*, the repeat tree is in agreement with trees based on plastid/plastome data (Fig. 3a; Rønsted et al. 2005; Day et al. 2014). The ML tree is partially resolved (Online Appendix 2). Owing to the huge genome sizes in this genus our analysis was based on a very low GP of 0.01%, and this may have had an impact on the tree building method—this level of GP should be sufficient, although it is on the cusp of being too low to be representative of repeat diversity and composition (Fig. 4a; see discussion below). However, it still reproduces species relationships in a similar manner to previous results, indicating that this number of reads still contains phylogenetic signal despite their low GP.

### Example 3—Drosophila (Drosophilidae; Diptera)

Our analyses for the diverse fly genus *Drosophila* focused on seven species from the *melanogaster* subgroup. *Drosophila simulans* and *D. sechellia* are strongly supported as sister species, to which *D. melanogaster* is then sister (Fig. 3b). *Drosophila suzukii* and *D. biarmipes* form a clade, which is sister to the *D. melanogaster* clade. *Drosophila ananassae* is sister to the rest, with rooting on *D. bipectinata*. These results (Fig. 3b) mirror those found in many recent phylogenetic studies based on large amounts of sequence data including mtDNA and nuclear markers (Obbard et al. 2012; Yang et al. 2012; Seetharam and Stuart 2013). The ML analysis mirrors these results with reasonably high levels of support (Online Appendix 2).

### Example 4—Asclepias (Apocynaceae; Gentianales)

To test our method on a difficult phylogenetic problem we investigated the Sonoran Desert clade (SDC) of *Asclepias* and present for comparison the rDNA and plastome results of Straub et al. (2012), with *A. syriaca* as the outgroup. In the repeat tree, *A. macrotis*, *A. coulteri* and *A. leptopus* are supported as separate from the core SDC, which includes *A. albicans*, *A. subulata*, *A. subaphylla*, *A. masonii* and *A. cutleri*; in plastid and nrDNA analyses a strongly supported core SDC excludes *A. cutleri* (Fig. 3c). Otherwise the results are different from both rDNA and plastomes, which are in turn different from one another and that from mtDNA (Straub et al. 2012). Note that species are not monophyletic (as with the plastome tree), and a putative homoploid hybrid was included (*A. albicans*, *A. subulata*). Thus our method provides yet another novel hypothesis of relationships between species for this difficult phylogenetic problem, although these relationships are weakly supported. It should be noted however that our results may be compromised by a lack of genome size data with which to calibrate the number of input reads. If there are large GS differences between species, then the GP analyzed will be significantly different between species, which may influence the topology of the trees produced.

### Example 5—Orobanchaceae (Lamiales)

The tree is rooted with *Lindenbergia philippensis* based on the analyses of Park et al. (2008) and Piednoël et al. (2012). *Orobanche* and *Phelipanche* are each monophyletic (Fig. 3d), and *Schwalbea americana* is then sister to them, generally exhibiting similar to greater levels of divergence than analyses based on DNA sequence data (Fig. 3d). Such high levels of divergence are only apparent in this dataset.

Internal relationships in *Orobanche* and *Phelipanche* are entirely congruent with previous analyses based on nrITS and plastid *rps2* sequence data, in all cases with similar or better support in our analysis (Fig. 3d; Schneeweiss et al. 2004; Park et al. 2008; Piednoël et al. 2012). The position of *O. gracilis*/*O. cumana* is switched, however, relative to the tree generated from full plastome sequences (Fig. 3d). The high support for the position of *O. gracilis* and the fact that there is no evidence of reticulation (result not shown) are congruent with an autopolyploid origin for this taxon. This could, however, reflect processes of diploidization and genome downsizing in an older allotetraploid, as shown in *Nicotiana* allopolyploids (Example 1); our result could also be affected by low taxon sampling within *Orobanche* (i.e., only one parent present). This might explain the difference between our placement and the one based on plastomes.

### Example 6—Tribe Fabeae (Fabaceae; Fabales)

Following the tree of Schaefer et al. (2012), our tree, with section *Ervilia*, now proposed to be genus *Ervilia* (*Vicia hirsuta*, *V. sylvatica,* and *V. ervilia*) as the outgroup, shows a closer relationship between *V. ervilia* and *V. sylvatica*. *Vicia tetrasperma* is the sole representative of section *Ervum* (proposed genus *Ervum*), which is sister to both *Pisum* and *Lathyrus* (Fig. 3e). The type section of *Vicia* is not included in this analysis. *Pisum* and *Lathyrus* are sister taxa, and *L. sativus* is sister to *L. vernus* and *L. latifolius*, a grouping that is incongruent with the weakly supported results of the DNA sequence data, in which *L. sativus* and *L. latifolius* are more closely related (Fig. 3e; Schaefer et al. 2012). The MP analysis generally has lower support than in other examples, but this broadly mirrors levels of support found in results based on nuclear nrITS and plastid markers (Schaefer et al. 2012). The ML analysis recovers some of the main groupings; however, many nodes collapse, and overall the ML tree is unresolved (Online Appendix 2). An alternative explanation for the lower levels of support found in this example is that perhaps in this case we are looking at a group of more distantly related taxa (comprising several relatively distant related genera), which may be approaching the limits of phylogenetic utility for repetitive DNA. The other examples may well be more closely related, but this is an area in which more investigation is needed to clarify the issues. Compared to the DNA sequence tree, the repeat tree has shorter internal branches and longer external branches, indicating that clustering information may be limited. However deep coalescence and/or extensive reticulation combined with many unsampled taxa may be the underlying problem in this complicated group of legumes. This may explain the poor resolution in Schaefer et al. (2012) as well as difficulties in our analyses.

### Evaluating Method Performance

Based on resampling the diploid *Nicotiana* data, several aspects of method performance were evaluated. At low GPs, 0.005–0.040%, trees lack resolution, and groupings are often inconsistent with those inferred from sequence data (Fig. 4a). Additionally, variance is greater at lower levels of GP, making the method less reliable. Above a GP of 0.1%, clustering and phylogenetic inference appear to be consistent and robust in comparison to trees derived from DNA sequence data. With these GPs, the number of clusters necessary to resolve the tree with high support is approximately 150 (Fig. 4b). With lower numbers of clusters (e.g., 5–45), the tree is either topologically inconsistent with trees inferred from sequence data or simply unresolved.

The matrices were then explored using partitions of 150 clusters to test how the phylogenetic signal varied across a large range of cluster abundances, with CL1 being the most abundant (Fig. 4c). There is no or little variance in phylogenetic signal across partitions when a suitably high GP has been used (e.g., 2%; Fig. 4c). However, when lower GPs are used, the signal degrades rapidly and randomly, particularly with low-abundance elements below cluster number 2000 (Fig. 4c). At a GP of 0.07% the trees produced become highly stochastic, either unresolved or showing inconsistent relationships with low support. At a lower GP the signal degrades more quickly as the data essentially become more quickly “coded” into presence/absence characters—the phylogenetic signal in the actual abundance of repetitive elements is entirely lost.

Range analysis showed remarkable similarity between trees produced from three independent samplings of 0.32% (as shown in the GP analysis above). The tree produced from the range of the mean cluster abundance ±1SE of the mean had a topology identical to the three samples as expected, and there are only minor branch length differences between all trees. Thus, there seems to be no significant advantage to undertaking such an analysis, although it may be advisable to do so given the ease of effort and proposed advantage of avoiding spurious groupings where species are not actually statistically different (i.e., they have overlapping normal distributions of cluster abundance).

**Figure 5.**
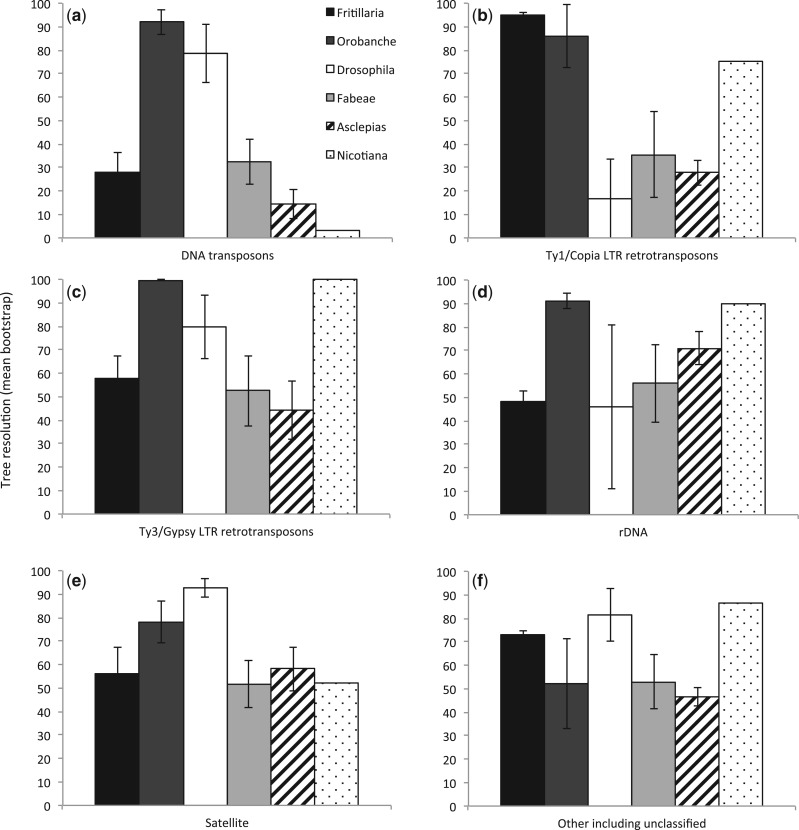
Impact of repeat type on tree resolution and method performance. Informativeness of each repeat type was estimated by creating subsets of the original matrices based on repeat annotation; in each case the mean bootstrap was calculated for each repeat type and each taxon dataset, error bars represent the standard error. a) DNA transposons. b) Ty1/Copia LTR retrotransposons. c) Ty3/Gypsy LTR retrotransposons. d) rDNA. e) Satellites. f) Other repeats including unclassified repeats and nonLTR retrotransposons.

Repeat types differed in their relative informativeness between datasets, but not in a consistent manner. Overall DNA transposons appeared to have less consistent phylogenetic signal than other types of repeats (Fig. 5). The relative informativeness of retrotransposons seems to be more taxon-specific, e.g., in some datasets Ty1/Copia were more informative than Ty3/Gypsy, whereas in others the opposite is true. The importance of including unclassified repeats is also highlighted by the informativeness of this category in all datasets examined.

## Discussion

### Resolving Species Relationships using Repeat Abundances

Using one insect and five angiosperm examples that vary in genome size by ∼400-fold (from 1C=0.2Gb in *Drosophila* to 75.7 Gb in *Fritillaria*), we have shown that using relative abundance of repetitive elements as continuous characters successfully resolves species relationships in a manner similar to that obtained by using DNA sequences from plastid and nuclear ribosomal regions. Using low coverage sequencing of genomic DNA (>0.1%GP) and ≥ 150 cluster abundances, we show that the repetitive DNA-based phylogeny reconstruction method is consistent in resolving expected relationships (i.e., those produced with other, more standard, methods, and data). The method can be seen as an additional source of phylogenetic information from the repetitive, noncoding portion of the genome, which will be a useful comparison to results based on DNA sequences. Gene trees represent the ancestry of particular sequences, and because allele histories may have differences from the species trees plus have coalescent times that differ from each other and species divergence times (Pillon et al. 2013) they often present conflicting topologies (Nichols 2001). Thus, there has been a recent focus on multiple sequence datasets, which aim to give a genome-wide assessment of species divergence. Here, our method provides data widespread across the genome, and each repeat abundance (cluster) is a marker; a matrix of such abundances likely represents many independently evolving characters. Furthermore at an appropriate level of GP there is little variance in phylogenetic signal across the dataset, showing remarkably consistency, although this requires further testing with additional datasets that include a larger number of taxa.

Our ML results on the whole agree with our maximum parsimony results, although for many groups the tree is only partially resolved, e.g., *Fritillaria* and tribe Fabeae (Online Appendix 2). We are unsure as to why ML does not perform as well as MP here, but the large state space of the character coding might provide insufficient information to accurately inform site likelihoods. We will develop further likelihood and Bayesian models for inferring trees from repeat abundances, but these approaches are limited by our understanding of repeat evolution, which is in its infancy. It is possible that horizontal transfer occurs for several repeats, but we believe the overall impact of this on our results is low, due to the large proportion of the genome covered by these analyses. Additional work is needed to further model the evolution of repeat populations, but we believe these results and others (e.g., Jurka et al. 2011,;012) provide evidence that repeats evolve primarily in accordance with random genetic drift; they therefore contain useful phylogenetic signal.

For reliable estimates of species relationships, we recommend using >0.1%GP for clustering and subsequent phylogenetic analysis based on at least 150 cluster abundances. If a lower GP is used we suggest using 1000 cluster abundances in the analysis for reliable detection of the phylogenetic signal present. With larger (and more repetitive genomes) it is possible to resolve relationships with lower levels of GP as read depth will likely still be sufficient at GP < 0.1%, as we observed here for *Fritillaria*.

### Why use Repeat Abundances for Phylogenetics?

There is an extensive literature on the evolution of repeats, see e.g., recent reviews by Kelly et al. (2012), Leitch and Leitch (2012), Kejnovsky et al. (2009), and Fedoroff (2012). All genomes contain tandem repeats and transposable elements, predominantly retrotransposons and derivative repeats—in plants these are typically long-terminal repeat (LTR) Ty1/Copia and Ty3/Gypsy elements (Hansen and Heslop-Harrison 2004). Copy number of these elements is highly variable and can change rapidly, contributing a large effect on genome size and architecture (Kelly and Leitch 2011). Genome content of these repeats, as a whole is the result of mechanisms of repeat expansion (e.g., retrotransposition, repeat recombination) and contraction (e.g., recombination-based deletion). In this study we show that the variable abundance of different repetitive elements contains phylogenetic signal, i.e., one reflecting the evolutionary history of these species, which would be expected, given the premise that repeats are an inherent structural feature of the genome and in fact underlie much of the evolution of large, complex eukaryotic genomes (Fedoroff 2012).

Previously, it has been shown that the frequency of short sequence repeats provide genomic signatures that can be used to reconstruct phylogenetic relationships (e.g., Edwards et al. 2002; Pride et al. 2003). Our approach builds on this insight to show that a genomic signature containing phylogenetic information extends to many larger repeat classes (Fig. 5), based on sequence similarity and graphical clustering. Potentially therefore it is likely to be robust to particular features of individual genomes being analyzed, which may or may not be rich in certain categories of repeat. Researchers using genome skimming approaches to assemble high-copy DNA features (plastomes, rDNA cistron) already have these repeat data available (e.g., Bock et al. 2014), and it provides another data source of noncoding nuclear DNA from which to infer phylogenetic hypotheses.

This method is genome-wide, and there is no need to attempt to distinguish paralogs, as essentially each cluster represents a homologous family of repeats (as determined by their highly conserved sequence, which is how they are clustered in the first place), the genomic abundance of which is used as phylogenetic data. In contrast, using low-copy nuclear markers requires paralogs to be distinguished accurately from one another. Low-copy nuclear genes are becoming increasingly popular due to often-higher variability when compared with plastid or mitochondrial markers (but not always, Turner et al. 2013), particularly for recent radiations and population studies. High variability can be seen as a consequence of having long coalescent times (compared to plastid or mitochondrial markers), but this in fact confounds their use, as when two or more young taxa share ancient alleles only due to the absence of fixation in ancestral populations rather than unique descent (Pillon et al. 2013). This means that many such markers are needed to provide evidence for which ones are providing spurious or conflicting results. Our repeat method, however, shows particular promise for these cases, as repeats evolve rapidly, and there is neither the added complication of comparing markers with different coalescent times nor effect of longer coalescent times and incomplete lineage sorting.

This method proved useful for inferring parents of some allopolyploid taxa in which extensive diploidization has occurred (i.e., replacement of repeats typical of one parent with those of the other) and repeat abundance is much more similar to one of the parents (here observed in groups of *Nicotiana* allopolyploids). In other groups, in which homoploid and polyploid hybridization occurs, further conflict will occur in construction of strictly bifurcating trees, but this can be analyzed more thoroughly using networks or pruning of putative parental or hybrid taxa. Using each cluster abundance (a single character) to make a separate estimate and then building a network based on these input trees, we might be able to extract in the repeat data further evidence of reticulation. Nonetheless, in our analyses repeat abundances have proven useful for determining one of the putative parents of allopolyploid taxa (Renny-Byfield et al. 2013), but perhaps with some refinement in methods we could be expected to demonstrate clear evidence for both parents, especially in recently synthesized hybrids (as in the *N. tabacum* analyses when we looked at individual trees from the bootstrap replicates). Additionally, this method has not yet been tested in a species or species complex of wide geographical range, which would be a useful further test for how this method performs at the intraspecific level. If homogenization of repeats occurs as a result of gene flow, which holds back formation of sequence variants, we predict that divergence occurs quickly once gene flow ceases, as might occur for isolated populations of a widespread species.

There is an additional caveat that should be mentioned; in order for the method to work reliably it is best that genome size is first estimated (via flow cytometry). If genome size is not used to standardize repeat abundances, then the resultant trees may reflect genome size differences more than shared evolutionary history, as repeat estimates reflect sampling bias rather than true abundance. It should be noted that the *Asclepias* example did not include genome size standardization as these data were not available. Additionally, we simulated lack of genome size information in the *Fritillaria* example, using the same number of reads (40 000) for each sample, despite >2-fold genome size differences (30–75 Gb), but results from this analysis closely mirror those presented in the results section, again with high levels of support (Online Appendix 3). Thus when genome sizes are unknown, the method still has the potential to produce a reliable phylogenetic result. It should therefore be possible to include data from various genomic sources, including available genome sequence data, although we do not know if this would vary relative to sequencing effort/coverage used in the clustering.

### Further Applications and Development of this Method

The proposed method may also prove useful for sequencing DNA from herbarium specimens, where genomic DNA is often degraded to a greater extent (Sarkiinen et al. 2012). Highly degraded DNA will be expected to contain intact copies of high-copy regions (i.e., particularly shorter repeats) even when other regions are largely eliminated. Herbarium specimens provide an invaluable source of plant material, often collected from remote regions and for taxa that may have since become rare or even extinct. Utilizing this resource will be a continued focus of research in plant systematics, and bridging the gap from Sanger sequencing of short markers to NGS of genome-wide markers is one current focus of research. Here, we provide one possible solution to this problem, as short NGS reads of low-coverage gDNA give us an invaluable insight into repetitive DNA proportions, a method that is advantageous because repeats are present in high-copy number and distributed across the genome. Unlocking and mining data from museum collections will understandably be a future focus of systematic studies (e.g., Guschanski et al. 2013).

The clustering pipeline utilized here has been shown to be effective in characterizing repetitive elements across various groups of eukaryotes including, for instance, bats (Pagan et al. 2012). Phylogenetic trees based on repeat abundances estimated with RepeatExplorer could be produced in other groups of animals, including mammals, and fungi. This makes the methodology particularly useful for those carrying out genome evolutionary studies in various types of organisms.

### Concluding Remarks

We conclude that this methodology is quick and easy to implement from the initial stage of DNA extraction and Illumina sequencing through to clustering of reads and tree building, utilizing the RepeatExplorer pipeline and TNT program (both freely available). We predict that as the cost of NGS continues to decrease in coming years (currently the major cost is in the library preparation), the overall cost of using this method will also decrease.

This method has proven successful in resolving species relationships as previously hypothesized by analyses of DNA sequence data (plastid and nuclear trees) and morphological circumscription in five diverse groups of angiosperms and one insect. There were only a few instances where the results are incongruent with trees derived from DNA sequences, for which evidence about cause should be sought; there are good reasons why in some cases plastid DNA and rDNA could be misleading, so these discrepancies should not be ignored or be thought of as a fault of using repeats as phylogenetic characters. This method does provide an important extension of molecular systematics methods and should be useful for comparative phylogenomics. At the same time as providing data for robust phylogenetic reconstruction in diploid species, this method provides abundant information for understanding genome evolution in the context of repetitive DNA. Indeed, this has already been done for a number of the datasets/partial datasets used in this study (e.g., Piednoël et al. 2012, 2013; Renny-Byfield et al. 2013).

## Supplementary Material

Data available from the Dryad Digital Repository: http://dx.doi.org/10.5061/dryad.vn0gc.
